# Tannin-Rich Chestnut and Persimmon Extracts in Puddings: Gelation, Proteins, and Antioxidant Activity

**DOI:** 10.3390/gels12020111

**Published:** 2026-01-27

**Authors:** Sae Kumagai, Tetsuya Takahashi, Yoko Tsurunaga

**Affiliations:** 1 Graduate School of Human and Social Sciences, Shimane University, Matsue 690-8504, Shimane, Japan; sae.kumagai@outlook.jp (S.K.); takahashi@hmn.shimane-u.ac.jp (T.T.); 2 Faculty of Human Science, Shimane University, Matsue 690-8504, Shimane, Japan; 3 The United Graduate School of Agricultural Sciences, Tottori University, 4-101 Koyama-Minami, Tottori 680-8553, Tottori, Japan

**Keywords:** sustainability, allergens, chestnut inner skin, young persimmon fruit, pudding gel, food gels, functional foods

## Abstract

To promote sustainable food production, the effective valorization of agricultural byproducts is essential. This study investigated the potential of underutilized chestnut inner skin (CIS) and thinned young persimmon fruit (YPF) extracts as functional ingredients in pudding gels, selected as a complex model system utilizing heat-induced egg gelation with milk and sugar. Puddings were prepared by replacing water with 10% or 50% CIS or YPF extracts. We comprehensively evaluated the physicochemical properties (texture, color, viscosity), microstructure (SEM), and sensory quality. Additionally, immunoreactive allergenic proteins (ovalbumin, casein, *β*-lactoglobulin) were quantified using ELISA, and antioxidant activity was measured via DPPH and H-ORAC assays. Results indicated that while high extract concentrations (50%) negatively impacted texture by increasing hardness and forming air pockets, the 10% YPF treatment yielded a smooth, homogeneous microstructure comparable to the control. Crucially, the 10% YPF extract significantly reduced the concentration of detectable allergenic proteins, attributed to the formation of insoluble tannin–protein complexes, without compromising sensory acceptance. Furthermore, the addition of these extracts significantly enhanced the antioxidant activity of the puddings in a concentration-dependent manner. These findings demonstrate that 10% YPF is a promising candidate for developing sustainable, hypoallergenic, and antioxidant-rich functional food products.

## 1. Introduction

In recent years, the generation of food-processing byproducts and agricultural waste has increased remarkably. To establish a sustainable social system, it is essential to develop technologies that enable the effective utilization of these materials [1]. In this study, we focused on two underutilized resources: the inner skin of chestnuts (*Castanea crenata*) and thinned young persimmon fruits (YPF) (*Diospyros kaki* Thunb.).

Chestnuts are widely used in processed products, such as glacé, syrup-cooked items, and pastes. However, substantial amounts of chestnut inner skin (CIS) are discarded during the manufacture of these products [2]. Similarly, YPF are typically removed during fruit thinning during cultivation and discarded in large quantities [3].

Both CIS and YPF are rich in tannins, a class of polyphenolic compounds [4,5]. Polyphenols are secondary plant metabolites characterized by aromatic rings bearing one or more hydroxyl groups [6,7,8]. According to Molino et al. [9], tannins are “water-soluble phenolic compounds with molecular weights between 500 and 3000 Da, that along with typical phenolic reactions, possess unique properties such as the ability to precipitate alkaloids, gelatin, and other proteins.” These properties highlight the protein-binding capacity of tannins.

Tsurunaga et al. [5] demonstrated that tannin-rich extracts from CIS and YPF enhanced the hardness of egg white gels. Moreover, the addition of tannin extract reduced the concentration of egg white allergen, which was attributed to the binding and aggregation of tannins with egg white proteins. That study significantly contributes to our understanding of the interaction between CIS/YPF extracts and proteins, demonstrating their effects on both physical properties (e.g., gel hardness) and functional aspects (e.g., allergen reduction). However, these findings were derived from simplified models. Whether similar effects are observed in complex food matrices that more closely resemble real-world conditions, where egg and milk proteins coexist with sugars, remains to be determined.

Egg white gels are rarely consumed in isolation. Therefore, processed egg-based products containing additional ingredients should be evaluated. In this study, we selected pudding, a representative gel-like food containing egg and milk proteins, as a model system and incorporated tannins in the form of CIS or YPF extracts.

Pudding is a hydrogel food composed of ingredients such as eggs, milk, and sugar, and its gel structure contributes significantly to its palatability [10]. The primary components of the gel structure are proteins derived from eggs and milk [11]. The major egg proteins are ovalbumin (OVA) and ovomucoid (OVM). OVA constitutes approximately 54% of egg white protein and is a major allergen; however, its allergenicity usually decreases upon heating or digestion [12]. OVM, which accounts for approximately 11% of egg white proteins, is the most allergenic component. Its stability is maintained by its robust tertiary structure and glycosylation, which make it relatively resistant to heat and digestive enzymes [13].

The principal milk proteins, casein (CAS) and *β*-lactoglobulin (*β*-LG), are also recognized as food allergens [14]. CAS is a major allergen in milk, and nearly all individuals with cow’s milk allergy exhibit specific reactions to it [15]. *β*-LG is also a major milk protein allergen, and heating above 65 °C alters its structure and reduces its allergenicity [16].

Therefore, the primary aim of this study was to valorize underutilized CIS and YPF by utilizing their tannin-rich extracts in pudding processing, thereby expanding their industrial applications. Specifically, we aimed to comprehensively evaluate the effects of these extracts on the physicochemical properties, gel microstructure, antioxidant activity, and sensory quality of the puddings. A key objective was to determine whether adding these extracts could effectively reduce the immunoreactivity of major egg and milk allergens (OVA, CAS, and *β*-LG) while maintaining consumer acceptance, thus establishing a model for developing functional, hypoallergenic foods from food by-products.

## 2. Results and Discussion

### 2.1. Characteristics of Pudding Materials

**Figure 1:**
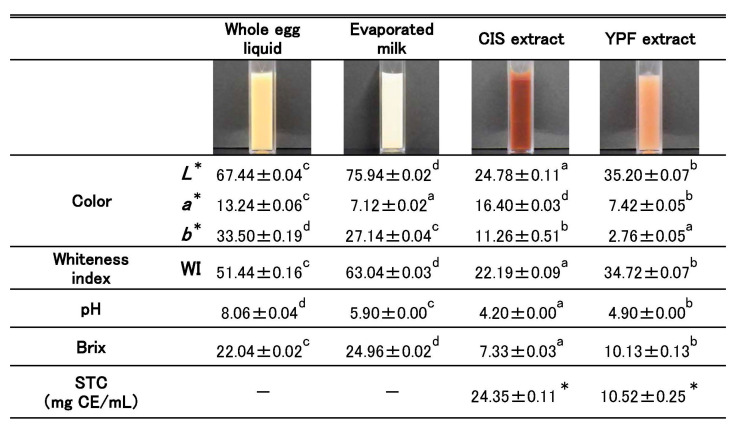
Color, pH, Brix, and soluble tannin content (STC) of the pudding ingredients. Color, whiteness index (WI), pH, and Brix values are expressed as mean ± standard error (*n* = 5). Different letters indicate statistically significant differences (*p* < 0.05). STC values are expressed as mean ± standard error (*n* = 4). Asterisks (*) indicate a significant difference between the groups, as determined by a *t*-test (*p* < 0.05). CIS extract, chestnut inner skin extract; YPF extract, young persimmon fruit extract; CE, catechin equivalent.

Figure 1 presents the color, whiteness index (WI), pH, and Brix values of the pudding ingredients (whole egg liquid, evaporated milk, and CIS and YPF extracts), as well as the soluble tannin content (STC) of the CIS and YPF extracts.

#### 2.1.1. Color

The L*, a*, and b* values differed significantly among the materials. The L* (lightness) and b* (yellowness) values of the whole eggs and evaporated milk were higher than those of the two extracts. Specifically, the L* (lightness) and b* (yellowness) values were the lowest for the CIS and YPF extracts, respectively. The a* (redness) value was highest for the CIS extract. Consequently, the CIS extract appeared reddish, yellowish, and dark. In contrast, the YPF extract was lighter than the CIS extract, and its redness and yellowish tones were weaker. The WI of the YPF extract was greater than that of the CIS extract.

#### 2.1.2. pH

The pH values of the whole egg and evaporated milk were 8.06 and 5.90, respectively. The pH values of the CIS and YPF extracts were 4.20 and 4.90, respectively, indicating that the extracts were slightly acidic.

#### 2.1.3. Brix

Whole egg liquid and evaporated milk exhibited higher Brix values compared to the CIS and YPF extracts. Notably, the Brix value of the YPF extract was significantly higher than that of the CIS extract.

#### 2.1.4. STC of CIS and YPF Extracts

The CIS extract had more than twice the STC of the YPF extract, indicating a considerable difference.

### 2.2. Pudding Quality Evaluation

#### 2.2.1. Stereomicroscopic Observation of Bubbles in Pudding Mixture

**Figure 2:**
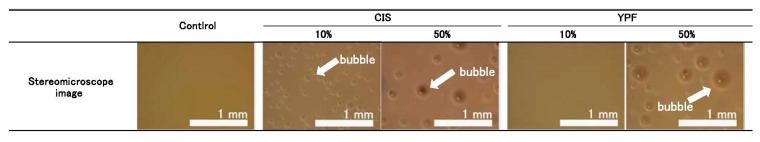
Stereomicroscopic images of pudding mixtures (magnification: 15×). Arrows indicate the bubbles in the pudding mixtures. CIS, chestnut inner skin; YPF, young persimmon fruit. Control refers to pudding mixture without CIS or YPF. Samples labeled 10% and 50% indicate pudding mixtures where the respective percentage of distilled water (50 g) was replaced with CIS or YPF extract.

Figure 2 shows stereomicroscopic images of the pudding mixtures. Bubbles were observed in the 10% CIS, 50% CIS, and 50% YPF samples.

#### 2.2.2. Static Viscosity of Pudding Mixtures

Table 1 presents the static viscosity of the pudding mixtures. Static viscosity increased significantly in the 10% CIS, 50% CIS, and 50% YPF samples compared to the control. In contrast, the 10% YPF sample did not differ significantly from the control.

#### 2.2.3. Surface Appearance and Physicochemical Properties of Pudding Gels

**Figure 3:**
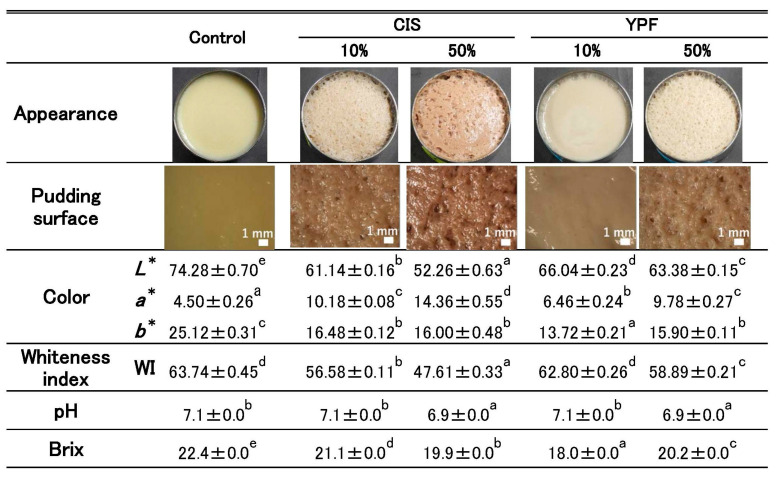
Surface images of pudding gels and their color, WI, pH, and Brix values. Digital photographs and stereomicrographs show the pudding surfaces. L*, a*, and b* refer to the CIE L*a*b* color space. Values are expressed as mean ± standard error (*n* = 5 for color, WI, and Brix; *n* = 3 for pH). Different letters indicate significant differences (*p* < 0.05). CIS, chestnut inner skin; YPF, young persimmon fruit. Control refers to pudding gel without CIS or YPF. Samples labeled 10% and 50% indicate pudding gels where the respective percentage of distilled water (50 g) was replaced with CIS or YPF extract.

Figure 3 shows digital photographs and stereomicroscopic images of the pudding gel surface, as well as the results of color, WI, pH, and Brix.

The surfaces of the control and 10% YPF pudding gels appeared smooth. In contrast, the surfaces of the 10% CIS, 50% CIS, and 50% YPF pudding gels were rough, with the 50% CIS gel specifically exhibiting large cavities.

Regarding color properties, the control pudding recorded the highest L* (lightness) and b* (yellowness) values, along with the lowest a* (redness) value. The L* values decreased, and the a* values increased with increasing amounts of CIS and YPF extracts in the pudding. The b* values increased significantly with increasing YPF extract content. No significant difference was observed in WI between the control and 10% YPF groups.

The pH values of all treatments were neutral, and the Brix value was the highest in the control pudding among all puddings.

#### 2.2.4. Cross-Sectional Structural Observation of Pudding Gels

**Figure 4:**
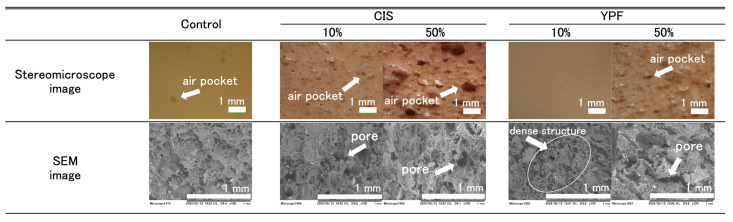
Cross-sectional images of the pudding gels. The pudding gel was observed using a stereomicroscope. SEM observations were performed at 100× magnification. The “dense structure” indicates a dense gel with small pores. CIS, chestnut inner skin; YPF, young persimmon fruit. Control refers to pudding gel without CIS or YPF. Samples labeled 10% and 50% indicate pudding gels where the respective percentage of distilled water (50 g) was replaced with CIS or YPF extract.

Figure 4 shows the stereomicroscopic and scanning electron microscope (SEM) images of pudding gel cross-sections.

Stereomicroscopic images showed that the control pudding gel had air pockets as large as 0.5 mm in diameter, but they were not uniform. Air pockets as small as 0.3 mm in diameter were observed in the 10% CIS and 50% YPF pudding gels. Extremely small air pockets were uniformly present throughout the 10% YPF pudding gel; however, they were difficult to observe. There were numerous air pockets in the 50% CIS pudding gel, some of which were as large as 1 mm in diameter.

The SEM images of the pudding gels with 10% CIS, 50% CIS, and 50% YPF showed irregular pores of varying sizes. In contrast, the pudding gel supplemented with 10% YPF exhibited small pores and a dense structure.

#### 2.2.5. Density of Pudding Mixtures and Gels

Table 2 lists the densities of the pudding mixtures and the gels. In the pudding mixtures, no significant differences were observed among the control, 10% CIS, and 10% YPF groups. However, a significant decrease was observed in the 50% CIS and 50% YPF groups. In the pudding gels, significant differences were observed among the samples, and the 10% YPF gel was denser than the control.

#### 2.2.6. Soluble Tannin Content and Antioxidant Activity of Pudding Gels

**Figure 5:**
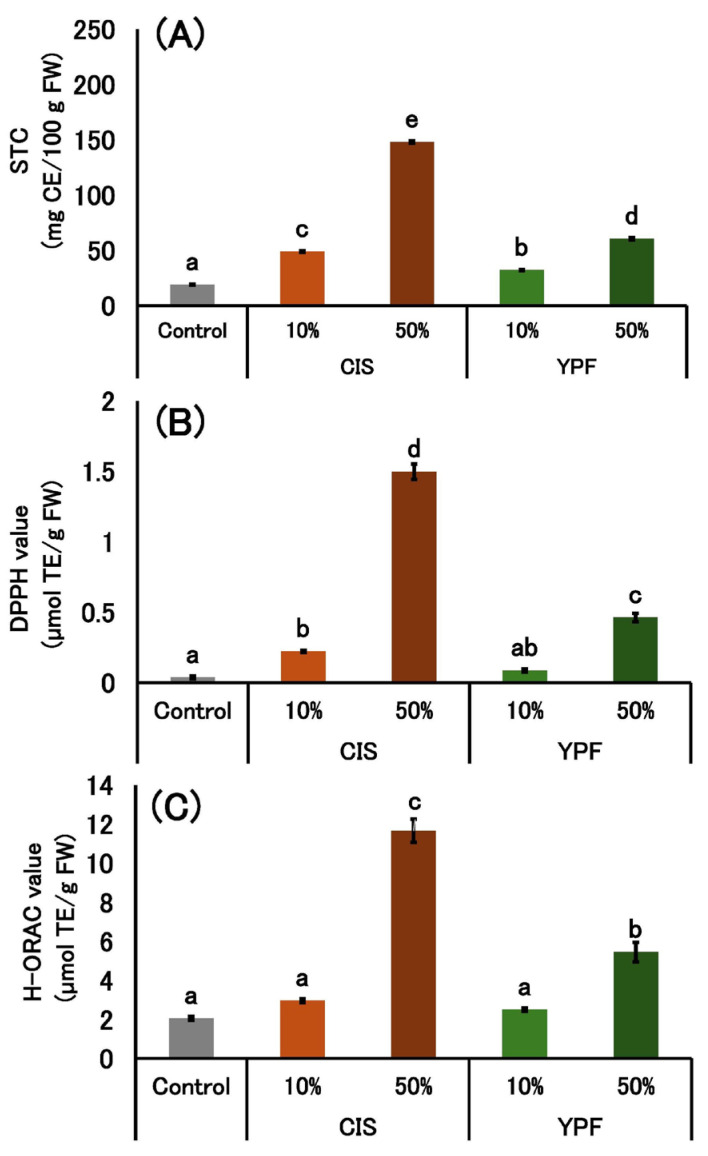
Soluble tannin content and antioxidant activity of pudding gels. (**A**) soluble tannin content (STC), (**B**) DPPH and (**C**) H-ORAC value are expressed as mean ± standard error (*n* = 5). Different letters in the figure indicate significant differences (*p* < 0.05). CIS, chestnut inner skin; YPF, young persimmon fruit; CE, catechin equivalent; TE, Trolox equivalent; FW, fresh weight. Control refers to pudding gel without CIS or YPF. Samples labeled 10% and 50% indicate pudding gels where the respective percentage of distilled water (50 g) was replaced with CIS or YPF extract.

Based on the STC of the CIS and YPF extracts, the theoretical soluble tannin content per 100 g of pudding mixture was calculated as 81.2 mg for 10% CIS, 405.8 mg for 50% CIS, 35.0 mg for 10% YPF, and 175.3 mg for 50% YPF. Figure 5 shows the measured STC and antioxidant activity (2,2-diphenyl-1-picrylhydrazyl (DPPH) and Hydrophilic Oxygen Radical Absorbance Capacity (H-ORAC) values) of the pudding gels. The measured STC values in the pudding gels were considerably lower than the theoretical values derived from the extracts.

Regarding antioxidative properties, both DPPH and H-ORAC values significantly increased in the puddings containing CIS and YPF extracts compared to the control. These activities exhibited a concentration-dependent increase, with the 50% CIS and 50% YPF samples showing the highest values.

#### 2.2.7. Texture Measurement of Pudding Gels

**Figure 6:**
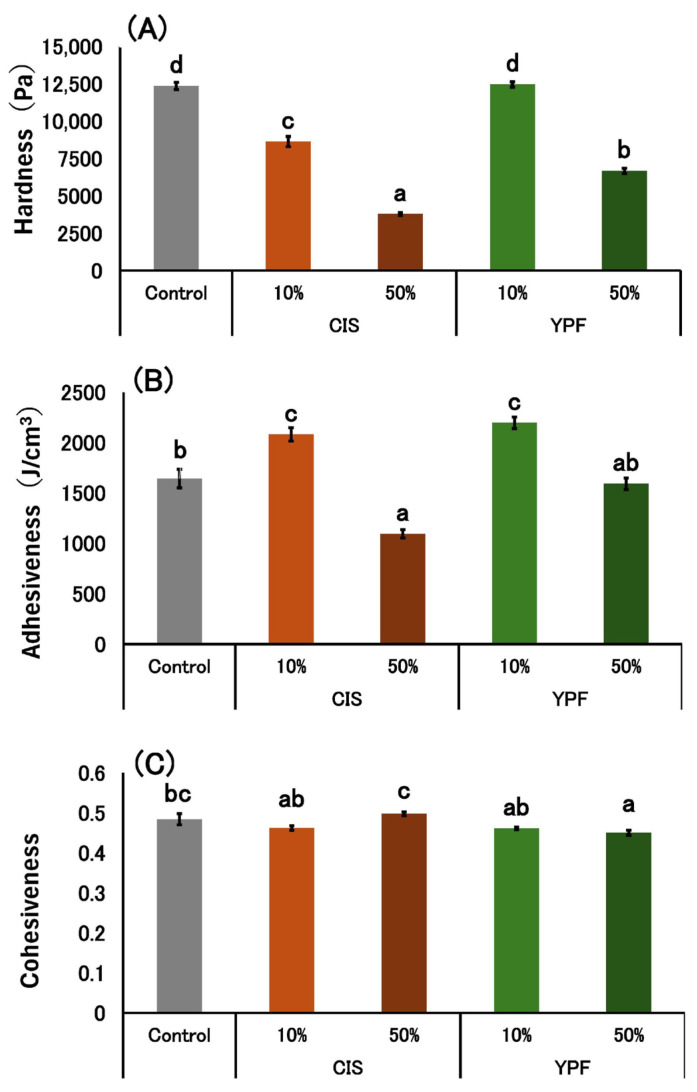
Pudding gel (**A**) hardness stress, (**B**) adhesiveness, and (**C**) cohesiveness. Texture is expressed as mean ± standard error (*n* = 5). Different letters in the figure indicate significant differences (*p* < 0.05). CIS, chestnut inner skin; YPF, young persimmon fruit. Control refers to pudding gel without CIS or YPF. Samples labeled 10% and 50% indicate pudding gels where the respective percentage of distilled water (50 g) was replaced with CIS or YPF extract.

Figure 6 shows the textural properties of the pudding gels. The hardness stress of the pudding gels decreased as the amount of CIS and YPF extracts increased; however, no significant difference was observed between the 10% YPF sample and the control (Figure 6A). The adhesiveness of the 10% CIS and 10% YPF pudding gels was significantly higher than that of the control, whereas the adhesiveness of the 50% CIS pudding gel was significantly lower than that of the control (Figure 6B). The cohesiveness of the 50% YPF gel was significantly lower than that of the control, but the cohesiveness of the other samples containing the extracts was not significantly different from that of the control (Figure 6C).

#### 2.2.8. Sensory Analysis

**Figure 7:**
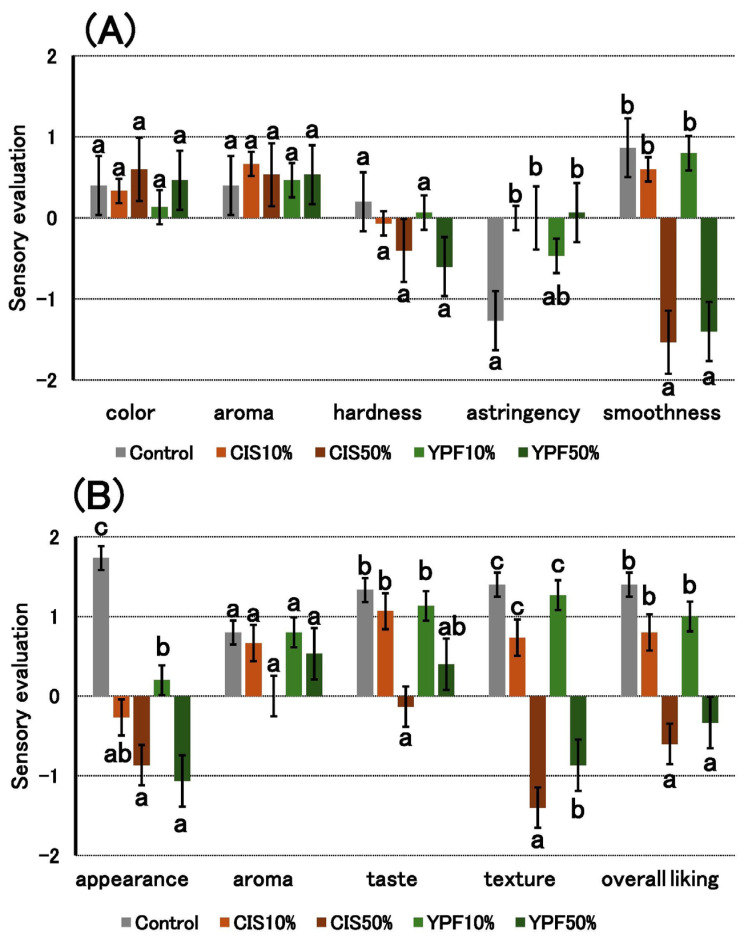
Sensory evaluation of the pudding gels. (**A**) Analytical test and (**B**) preference test scores are shown as mean ± standard errors on a 5-point scale (−2 = weak/unfavorable, 2 = strong/favorable) (*n* = 15). Different letters in the figure indicate significant differences between the five treatments for the same evaluation item (*p* < 0.05). CIS, chestnut inner skin; YPF, young persimmon fruit; Control refers to pudding gel without CIS or YPF. Samples labeled 10% and 50% indicate pudding gels where the respective percentage of distilled water (50 g) was replaced with CIS or YPF extract.

Figure 7A presents the results of the analytical sensory test. No significant differences in “color” and “aroma” were detected among the puddings. The “hardness” of the 50% CIS and 50% YPF puddings were perceived as lower, but no significant difference was found compared to that of the control. Pudding gels containing CIS and YPF extracts were identified as normal in terms of astringency. Furthermore, the 10% YPF pudding gel was identified as less astringent than the other groups and showed no significant difference compared to the control. The “smoothness” of the 50% CIS and 50% YPF samples showed negative results. However, the smoothness of 10% CIS and 10% YPF did not differ significantly from that of the control, which had the highest smoothness.

Figure 7B presents the results of the preference test. The “appearance” of the control showed the highest value among all pudding gels, indicating that it was well liked. Among the CIS and YPF extract-added pudding gels, only the 10% YPF sample showed a positive value, whereas all other puddings showed negative values, indicating that their appearance was not preferred. The “aroma” did not differ significantly among the puddings. The “taste” was not significantly different between the control, 10% CIS, 10% YPF, and 50% YPF, indicating that these puddings were equally well liked. The “taste” of 50% CIS was significantly lower than that of the control. No significant differences were found in “texture” and “overall liking” among the control, 10% CIS, and 10% YPF, indicating that these were preferred similarly to the control. In contrast, the “texture” and “overall liking” scores of 50% CIS and 50% YPF were significantly lower than those of the control, indicating that they were not preferred.

### 2.3. Determination of OVA, CAS, and β-LG Concentrations in the Pudding Gels

**Figure 8:**
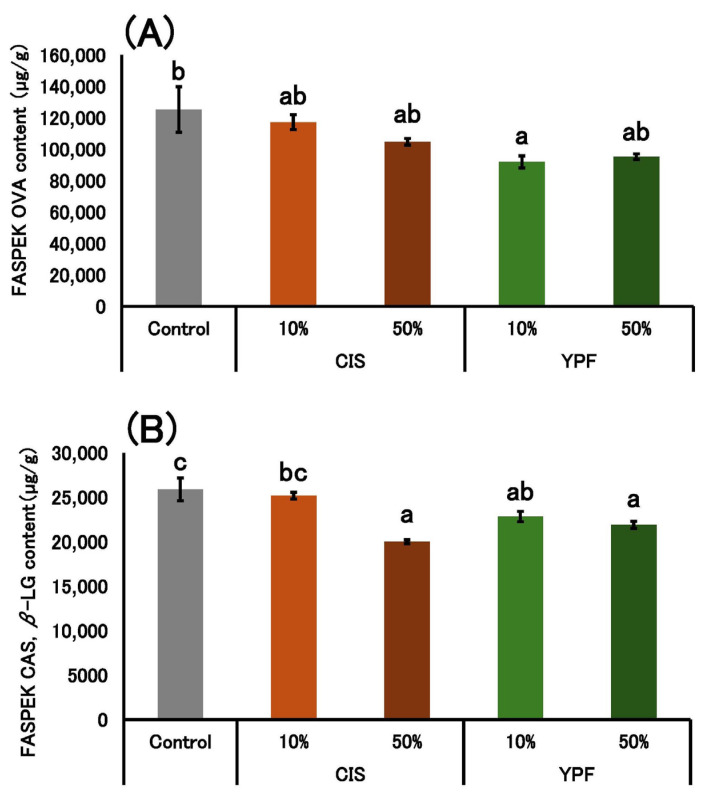
Allergen content of the pudding gels. The (**A**) OVA content and (**B**) CAS and *β*-LG contents of the pudding gels are shown as mean ± standard error (*n* = 4). Different letters in the figure indicate significant differences (*p* < 0.05). CIS, chestnut inner skin; YPF, young persimmon fruit; Control refers to pudding gel without CIS or YPF. Samples labeled 10% and 50% indicate pudding gels where the respective percentage of distilled water (50 g) was replaced with CIS or YPF extract.

Figure 8 shows the OVA, CAS, and *β*-LG concentrations in pudding gels. Compared with the control group, the OVA concentration in the 10% YPF group was significantly reduced. The total concentrations of CAS and *β*-LG in 50% CIS, 10% YPF, and 50% YPF groups were also significantly lower than those in the control.

### 2.4. Discussion

In this study, we investigated the effects of adding CIS and YPF extracts on the quality of pudding gels (appearance, gel structure, texture, and taste). The results indicated that the 10% YPF sample maintained a quality comparable to that of the control. The Brix value of the YPF extract was higher than that of the CIS extract (Figure 1). We attribute this result to the fact that YPF, which is a fruit, contains more sugar than the inner skin, CIS.

Figure 3 shows that the L* and a* values of the pudding gel surface in the 10% YPF group were closest to those of the control, although they were significantly different. In contrast, the b* value of the 10% YPF sample was the lowest among all pudding gels. The b* value of the YPF extract was lower than those of the whole egg liquid and evaporated milk (Figure 1), suggesting that the addition of the YPF extract reduced the b* value of the pudding gels. The WI of the 10% YPF pudding gel was not significantly different from that of the control. The WI increases with increasing L* values and decreasing a* and b* values. This study indicated that the L* and a* values for the 10% YPF pudding gel were closest to those for the control, whereas the b* value was lower. Therefore, the WI values of the 10% YPF and control groups were considered similar.

The surface of the 10% YPF pudding gel was as smooth as that of the control (Figure 3). In the cross-sectional observation, the air pockets of the 10% YPF pudding gel were so small that they could not be seen in the stereomicroscopic images; furthermore, there were no irregularly sized air pockets, as seen in the control (Figure 4). Furthermore, the SEM images of the 10% YPF pudding gel showed no large pores and an overall dense gel structure (Figure 4), and its gel density was significantly higher than that of the control (Table 2). These results suggest that the 10% YPF gel was denser than the control. In contrast, the surfaces of the 10% CIS, 50% CIS, and 50% YPF pudding gels were rough, and large irregular air pockets were formed in their cross-sections (Figure 3 and Figure 4). Stereomicroscopic images of the gel surfaces showed that the size of the air pockets increased in the order of 10% CIS, 50% YPF, and 50% CIS. The mechanism of air pocket formation observed in this study is consistent with the review by Aghajanzadeh et al. [17], which indicates that during food processing, water contained in the food evaporates upon heating, and the air incorporated during mixing acts as nuclei for bubble and pore (referred to as “air pockets” in this study) formation. As heating progresses, these bubbles and pores enlarge further, significantly affecting the final structure and texture of the gel [17].

The proteins surrounding these bubbles tend to coagulate, resulting in the stabilization of air pockets within the food matrix. In this study, we assumed that a similar mechanism occurred in the pudding gels. Specifically, bubbles persisted in the pudding mixtures containing 10% CIS, 50% CIS, and 50% YPF (Figure 2), likely contributing to the formation of air pockets in the final pudding gels. Furthermore, the static viscosity of the pudding mixtures was significantly higher in the 10% CIS, 50% CIS, and 50% YPF samples than in the control (Table 1). This increase in viscosity may inhibit bubble escape, allowing them to remain in the mixture and subsequently form air pockets during gelation. Previous studies have reported that bubbles in low-viscosity liquids are unstable and collapse easily [18,19]. In contrast, bubbles formed in high-viscosity liquids are stabilized and less likely to collapse [20,21]. In this manufacturing process, bubbles are generated during mixing and are likely to be trapped by the increased viscosity of the pudding mixture. The coagulation temperature of egg proteins is 80–85 °C [22,23]. In this experiment, the 10% CIS, 50% CIS, and 50% YPF pudding gels exhibited many air pockets. This is likely because the bubbles were fixed within the coagulated gel when the pudding mixture reached this temperature during heating. While the retention of air bubbles provided evidence of increased viscosity due to tannin–protein interactions, eliminating these bubbles is desirable for industrial applications to achieve a smoother texture. Specifically, to mimic standard market products, defoaming techniques such as ultrasonic degassing can be employed prior to steaming [24,25]. This would allow for the production of smooth, bubble-free puddings despite the increased viscosity.

Based on the STC of the extracts (Figure 1), the theoretical soluble tannin content per 100 g of pudding mixture was calculated as 81.2 mg (10% CIS), 405.8 mg (50% CIS), 35.0 mg (10% YPF), and 175.3 mg (50% YPF). However, as shown in the results (Figure 5), the measured STC in the final gels was considerably lower than these theoretical values. This discrepancy suggests that a significant portion of the added tannins bound firmly to the egg and milk proteins, forming insoluble complexes that could not be extracted by the solvent. This strong protein–tannin interaction is likely the primary mechanism responsible for the observed reduction in immunoreactive allergen proteins, as well as the increased viscosity and bubble retention.

Specifically, the higher the theoretical tannin content, the more bubbles were retained in the pudding mixture. The 10% YPF sample contained the lowest theoretical tannin content (35.0 mg) per 100 g of pudding gel, showing no bubble formation and no significant difference in static viscosity compared to the control (Table 1). Although these results suggest that STC affects the static viscosity of the pudding mixture, the increase in static viscosity was not strictly dependent on the STC per 100 g of pudding. Therefore, we hypothesize that this depends on the components of CIS and YPF other than tannins; however, the reason for this remains unclear.

Furthermore, the addition of CIS and YPF extracts imparted antioxidant activities to the pudding gels. As shown in Figure 5, both DPPH and H-ORAC values increased in a concentration-dependent manner, with the 50% CIS and 50% YPF samples exhibiting the highest activity. Phenolic compounds, including tannins, are known to exhibit strong antioxidant activity. Although the measured STC was lower than the theoretical value due to protein binding, the significant increase in antioxidant activity compared to the control suggests that the added CIS and YPF extracts effectively enhanced the functional value of the puddings. This indicates that these puddings could potentially serve as a source of dietary antioxidants.

The hardness stress results of the texture measurement (Figure 6A) showed that the hardness of pudding gels other than the 10% YPF sample was significantly lower than that of the control, and the 50% CIS had the lowest hardness. This suggests that the larger the air pocket formation, the lower the hardness of the pudding gel, and that suppressing bubble formation is necessary to maintain the same level of hardness as that in the control. Therefore, we consider that the 10% YPF sample, which did not form air pockets, was superior in terms of maintaining pudding gel hardness, whereas the results of the analytical sensory evaluation item “hardness” showed no significant difference among the pudding gels (Figure 7A). In contrast, the results of the sensory evaluation items, “smoothness,” “texture,” and “overall liking” showed no significant difference among the 10% CIS, 10% YPF, and control groups (Figure 7). The 10% YPF pudding gel showed no significant difference in hardness compared to the control, but its adhesiveness was significantly higher (Figure 6B). Therefore, this pudding gel was evaluated as having the same level of “smoothness,” “texture,” and “overall liking” as that of the control. According to Elmore et al. [26], creaminess is required for the preferred vanilla pudding and is associated with smoothness. Therefore, we consider that a “smooth texture” free of air pockets is preferred for a good-tasting pudding.

The control and 10% YPF treatments, which exhibited no surface air pockets, were rated positively for “appearance” in the preference test (Figure 7B). Smooth appearance is also a factor that indicates the favorability of a pudding gel. In a study by Li et al. [27], preferred milk puddings were reported to have a smooth mouthfeel and surface, which is consistent with our results. The smooth appearance and texture of the 10% YPF sample suggest that its product quality can be maintained at the same level as that of the control. In this study, cohesiveness (Figure 6C) was not related to the STC in the pudding gel or bubble formation in the pudding mixtures. Furthermore, the adhesiveness of the 10% CIS and 10% YPF pudding gel increased significantly compared to that of the control (Figure 6B). Adhesiveness indicates the resistance force when the plunger is pulled out of the food. In this study, we suggest that when the plunger is pulled up from the pudding gel, the shape of the pudding returns to its original shape, and adhesiveness increases. Because we measured the pudding gel in a stainless-steel Petri dish, we assumed that the density and adhesiveness of the pudding increased. However, no correlation was observed between pudding density and adhesiveness. Selway and Stokes [28] reported that adhesiveness is correlated with the condition of food surfaces, and Foegeding et al. [29] demonstrated that smoother custard surfaces resulted in lower adhesiveness. However, the surface of the 10% YPF group did not differ from that of the control group. Therefore, we could not determine the factors responsible for these differences in adhesiveness results. In the future, we aim to consider various possibilities and study them in further detail.

The STC of the CIS extract was approximately twice that of the YPF extract (Figure 1). However, it is unlikely that only the amount of soluble tannin added to the pudding affects the bubble formation. This is because the binding of tannin-containing polyphenols to proteins affects their protein structure. In reviews by Quan et al. [30] and Hagerman and Butler [31], the binding of polyphenols and proteins mainly involves hydrogen bonding and hydrophobic interactions between the hydroxyl groups of polyphenols and the amino and hydroxyl groups of proteins. Although the structure of tannin in CIS remains to be clarified, the tannins in YPF are reported to be proanthocyanidin polymers with a molecular weight of approximately 10,000 and comprising approximately 30 molecules of condensed catechins [32]. Therefore, we hypothesize that the number of hydroxyl groups in the tannin structure may affect the ease with which tannins bind to proteins. The structure and polymerization of tannins in the CIS and YPF extracts need to be examined, along with the amount of tannin added and its quality.

A reduction in the concentrations of OVA, CAS, and *β*-LG compared to the control was observed in the 10% YPF group (Figure 8). Specifically, a significant decrease in OVA concentration compared to the control was observed only in the 10% YPF group. The concentrations of CAS and *β*-LG in the 50% CIS, 10% YPF, and 50% YPF groups were also significantly lower than those in the control. Tsurunaga et al. [5] reported that the amount of egg white allergen in egg white gel with tannin decreased depending on the amount of tannin added [5]. However, the decrease in OVA, CAS, and *β*-LG concentrations in the pudding gel was not dependent on STC. This may be because the binding affinity of tannins to proteins varies depending on the protein type. Hagerman and Butler [31] reported that proline-rich proteins have a high affinity for tannins. Kanakis et al. [33] reported that hydrophobic amino acids bind readily to polyphenols. More than half of the constituent amino acids of OVA, an egg protein quantified in this study, are hydrophobic amino acids [34], and CAS, a milk protein, is rich in proline [35]. Based on this, we speculate that OVA and CAS bind readily to tannins, and that tannins bind preferentially to these proteins. In contrast, *β*-LG, which contains many hydrophilic amino acids, is considered less likely to bind to tannins. This suggests that differences in tannin–protein binding affinities may play a role. From a hydrogel viewpoint, this reduction can be attributed to the formation of insoluble protein–polyphenol complexes within the gel matrix. Polyphenols in YPF, such as tannins, likely act as cross-linkers, binding to protein side chains via hydrogen bonding and hydrophobic interactions [36,37]. This interaction promotes protein aggregation and incorporation into a denser, more cross-linked hydrogel network, making the proteins less extractable in the soluble fraction used for quantification.

In this study, only OVA among egg proteins, and CAS and *β*-LG among milk allergens, could be quantified. However, OVM is also present in egg white protein and is a major allergen. OVM is not easily denatured by heat and has the highest allergenicity among all egg allergens [38]. Therefore, the OVM content of the pudding gels needs to be examined. Moreover, the reduction in allergen concentrations reported in this study is thought to be attributed to the binding of tannins to the proteins, which physically inhibits the binding of specific antibodies to the protein epitopes. For this effect to be clinically relevant, the binding state of tannins and food proteins must be maintained in vivo. However, since ingested food is subjected to digestive enzyme-mediated degradation before reaching the intestinal tract, the binding of tannins to food proteins during digestion requires further investigation. It should be noted that the sensory evaluation in this study was conducted with a limited number of panelists (*n* = 15), a sample size comparable to that used in similar preliminary studies on food gels [39]. While statistically significant differences were observed, confirming the validity of the comparisons within this group, a larger consumer panel will be necessary in future studies to generalize these findings to a broader market.

The results of this study indicated that adding 10% YPF extract to pudding gels could reduce the concentration of OVA (egg protein) and of CAS and *β*-LG (milk proteins), while maintaining a quality level comparable to that of the control. Moreover, the addition of these extracts significantly enhanced the antioxidant activity of the puddings, suggesting their potential value as functional foods. From an industrial perspective, the findings of this study suggest a promising model for utilizing other tannin-rich food by-products in pudding processing. By elucidating the optimal types and concentrations of tannins, it is possible to develop functional ingredients that significantly reduce allergenic proteins levels and offer health benefits such as antioxidant activities, while maintaining product quality, thereby expanding their versatility in the food industry. Future research should focus on the structural characterization of tannins in CIS and YPF, specifically determining their polymerization degree and binding affinities with specific proteins. Furthermore, it is essential to investigate the stability of tannin–protein complexes during digestion and absorption using in vitro and in vivo models, as well as to explore the application of these extracts to other processed food matrices.

## 3. Conclusions

Pudding gels incorporating CIS and YPF extracts were characterized by structural analysis, texture measurement, and sensory evaluation. Additionally, we investigated the reduction in OVA, CAS, and *β*-LG concentrations driven by tannin–protein interactions.

In this study, the 10% YPF gel exhibited surface whiteness and smoothness comparable to the control. Structural analysis revealed a dense internal network in the 10% YPF gel. Furthermore, sensory analysis showed no significant differences from the control in terms of smoothness, taste, texture, or overall liking, indicating high consumer acceptance.

Crucially, the quantification of protein concentrations confirmed that OVA, CAS, and *β*-LG levels were reduced in the 10% YPF group compared to the control. From a hydrogel viewpoint, this reduction can be attributed to the formation of insoluble protein–polyphenol complexes within the gel matrix. Polyphenols in YPF, such as tannins, likely act as cross-linkers, binding to protein side chains via hydrogen bonding and hydrophobic interactions [40,41]. This interaction promotes protein aggregation and incorporation into a denser, more cross-linked hydrogel network, making the proteins less extractable in the soluble fraction used for quantification.

These results suggest that 10% YPF can effectively reduce allergen concentrations while maintaining quality comparable to standard pudding. This study highlights the potential of utilizing agricultural byproducts to develop sustainable, hypoallergenic food products.

## 4. Materials and Methods

### 4.1. Materials

The inner skin of chestnuts (CIS; *Castanea crenata* cv. ‘Porotan’) was sourced from Tsukuba City (Ibaraki, Japan). After separating the skin from the fruit, the CIS was dehydrated at 60 °C for 12 h using an incubator (DN-61, Yamato Scientific Co., Ltd., Tokyo, Japan). Young persimmon fruits (YPF; *Diospyros kaki* cv. ‘Saijo’) were collected from the Shimane Agricultural Technology Center (Izumo, Shimane, Japan). The calyxes and seeds were removed prior to freeze-drying. Both dried CIS and YPF were pulverized into fine powders using a grinder (ABS-W, Osaka Chemical Co., Ltd., Osaka, Japan) and passed through a 1 mm mesh sieve to ensure uniformity.

Hot water extraction was selected to ensure food safety and to avoid the use of organic solvents, aligning with green chemistry principles. Hot water extracts of CIS and YPF were prepared following the protocol described in our previous study [5]. Briefly, 45 g of each powder was mixed with 300 mL of distilled water and autoclaved at 120 °C for 20 min (LSX-300, TOMY SEIKO Co., Ltd., Tokyo, Japan). The supernatants collected after centrifugation (1000× *g*, 3 min) were used as the tannin extracts.

### 4.2. Preparation of Pudding

Pudding samples were formulated using whole frozen eggs (Sanyo Egg Co., Ltd., Chiba, Japan), evaporated milk (MEGMILK SNOW BRAND Co., Ltd., Tokyo, Japan), and granulated sugar (Mitsui DM Sugar Co., Ltd., Tokyo, Japan). The base mixture consisted of whole egg liquid (50 g), granulated sugar (15 g), and evaporated milk (35 g). To this base, 50 g of liquid was added: distilled water for the control, or aqueous tannin extracts (containing 24.35 mg catechin equivalent (CE)/mL for CIS and 10.52 mg CE/mL for YPF) replacing 10% (5 g) or 50% (25 g) of the water volume for the test samples. These five variations were designated as Control, 10% CIS, 50% CIS, 10% YPF, and 50% YPF.

Preparation was carried out by first blending the evaporated milk with distilled water or the respective tannin extract for 1 min using a mixer (Osterizer 16-speed blender, Sunbeam Oster, Boca Raton, FL, USA). Subsequently, the whole egg liquid and sugar were incorporated and blended for another minute. The resulting mixture was dispensed into stainless-steel Petri dishes (40 mm diameter × 15 mm height; ST-40, Yamaden Co., Ltd., Tokyo, Japan) and heated in a steam oven (NE-BS1600, Panasonic Corporation, Tokyo, Japan) at 90 °C for 15 min. The samples were left to set using residual heat for 3 min, followed by cooling in a refrigerator for at least 1 h.

To determine the concentrations of allergenic proteins (OVA, CAS, and *β*-LG) using specific ELISA kits (as detailed in Section 4.5), the prepared puddings were lyophilized using a freeze dryer (Alpha1-2LDplus, Martin Christ Gefriertrocknungsanlagen GmbH, Osterode, Germany) to obtain a freeze-dried powder (FD powder).

### 4.3. Characterization of Pudding Materials

#### 4.3.1. Measurement of Color, pH, and Brix

The chromatic attributes of the pudding and its raw ingredients (whole egg, evaporated milk, and CIS/YPF extracts) were evaluated based on the CIE L*a*b* color system. Samples were dispensed into stainless-steel Petri dishes, and surface color was measured using a color reader (CR-13, Konica Minolta, Inc., Tokyo, Japan). Prior to the measurements, the instrument was calibrated using a standard white calibration plate supplied by the manufacturer. The color parameters were expressed as L* (lightness), a* (red–green), and b* (yellow–blue). Furthermore, based on these parameters, the Whiteness Index (WI) was derived using Equation (1) [42].

(1) $$WI=100-\sqrt{(100-L{)}^{2}+a^{2}+b^{2}}$$

Additionally, pH levels and soluble solids (Brix) were determined using a pen-type pH meter (DPH-2, Atago Co., Ltd., Tokyo, Japan) and a pocket saccharimeter (APAL-J, AS ONE Corp., Osaka, Japan), respectively. Measurements were performed in quintuplicate for color and pH (*n* = 5) and in triplicate for Brix (*n* = 3); the mean values were used for analysis.

#### 4.3.2. Density and Soluble Tannin Content of the Extracts

The density of the CIS and YPF extracts was determined by measuring the mass of a 10 mL aliquot in a graduated cylinder and calculating the mass-to-volume ratio [43]. Measurements were performed in triplicate, and the mean values were recorded.

The soluble tannin content (STC) was quantified using the Folin–Ciocalteu method. While this method typically measures total phenolic content, existing literature indicates that the polyphenols in these extracts are predominantly tannins. According to Pinto et al. [2], as well as studies by Tsujita et al. [44] and Squillaci et al. [45], CIS polyphenols are primarily condensed tannins (prodelphinidins) composed of gallocatechin and catechin units esterified with gallate. Similarly, YPF polyphenols are characterized as proanthocyanidin polymers, which are also classified as condensed tannins [32]. Furthermore, previous studies have validated the utility of the Folin–Ciocalteu method for assessing STC in YPF and CIS [46,47]. Based on these collective findings, we considered the polyphenolic fraction in CIS and YPF to be predominantly tannins, with negligible low-molecular-weight polyphenols. Consequently, the STC was determined via the Folin–Ciocalteu assay, and the results were expressed as mg catechin equivalent per mL (mg CE/mL).

### 4.4. Pudding Mixture and Gel Quality Evaluation

#### 4.4.1. Stereomicroscopic Observation of Bubbles in the Pudding Mixture

To assess the air voids prior to heating, the pudding mixture was dispensed into a disposable cell (standard type, AS ONE Corp., Osaka, Japan), which was then sealed to prevent leakage. The cell was positioned horizontally, and the internal bubbles were observed using a stereomicroscope (DS-L3, Nikon Solutions Co., Ltd., Tokyo, Japan) at a magnification of 15×. In this study, voids observed in the sol state (before heating) are defined as “bubbles”, whereas those present in the gel state (after heating) are referred to as “air pockets”.

#### 4.4.2. Determination of Static Viscosity

The viscosity of the pudding mixtures was assessed using a sinewave vibroviscometer (SV-10, A&D Company, Ltd., Tokyo, Japan). This instrument employs a tuning-fork vibration method, where the driving force required to maintain a constant vibration amplitude of the oscillator plates is proportional to the product of the liquid’s viscosity (*η*) and density (*ρ*) (2) [48]. The measured value is defined as “static viscosity” (*sv*), calculated as:

*sv* = *η* × *ρ* (2)

A 10 mL aliquot of each mixture was transferred to the specified sample cup, and measurements were conducted at a controlled temperature of 25 ± 2 °C. To calculate the static viscosity, the stable reading recorded after 15 min was divided by the density of the respective pudding mixture. All measurements were performed in triplicate (*n* = 3), and the results are presented as mean values.

#### 4.4.3. Observation of Pudding Gel Appearance and Surface, and Measurement of Color

The macroscopic appearance and surface microstructure of the pudding gels were documented using a digital camera and a stereomicroscope (DS-L3, Nikon Solutions Co., Ltd., Tokyo, Japan), respectively. Regarding chromatic attributes, the surface tone was evaluated based on the CIE L*a*b* system, and the WI was derived from the measured coordinates. Color measurements were performed in quintuplicate (*n* = 5) for each treatment, and the mean values were calculated.

#### 4.4.4. Microstructural Analysis of Pudding Gels

The internal microstructure of the pudding gels was characterized using both a stereomicroscope and a table scanning electron microscope (SEM; TM3000, Hitachi High-Tech Corp., Tokyo, Japan) without conductive coating (sputtering). For stereomicroscopic observation, the gels were removed from the Petri dishes and cross-sectioned using a surgical scalpel to expose the internal surface. For SEM analysis, specimens measuring approximately 20 × 5 × 5 mm were excised from the central portion of the gel. Fixation was performed according to the protocol described by Deng et al. [49]; briefly, samples were immersed in a 2.5% (*v*/*v*) glutaraldehyde solution in 0.1 M phosphate buffer (pH 7.2) for 24 h. Following fixation, the specimens were rinsed three times with phosphate buffer and subsequently freeze-dried. The dried samples were mounted on SEM stubs using double-sided carbon tape (Nisshin EM Co., Ltd., Tokyo, Japan) and observed at a magnification of 100×. The detailed observation conditions were as follows: accelerating voltage, 15.0 kV; working distance, 6.1 mm; emission current, 24.9 µA; and observation mode, low vacuum (charge-up reduction mode).

#### 4.4.5. Determination of the pH and Brix of Pudding Gels

The pH levels were assessed by inserting the electrode of a pen-type pH meter (DPH-2, Atago Co., Ltd., Tokyo, Japan) directly into the pudding gel samples. Regarding soluble solids (Brix), the pudding gel was diluted with an equal volume of distilled water, and the resulting mixture was analyzed using a pocket saccharimeter (APAL-J, AS ONE Corp., Osaka, Japan). The final Brix content was calculated by multiplying the measured value by a dilution factor of 2. Measurements were conducted in triplicate for pH (*n* = 3) and in quintuplicate for Brix (*n* = 5); the results are reported as mean values.

#### 4.4.6. Density Determination for Pudding Mixtures and Gels

The density of the pudding mixture (sol state) was assessed following the same protocol described in Section 4.3.2. For the pudding gels, density was calculated based on the mass-to-volume ratio. The effective volume of the stainless-steel Petri dish was calibrated using distilled water and determined to be 18.7 cm^3^. The density was derived by dividing the mass of the gel within the dish by this volume. All measurements were performed in triplicate (*n* = 3), and the results are reported as mean values.

#### 4.4.7. Texture Analysis

The textural properties of the pudding gels were characterized directly in the stainless-steel Petri dishes using a creep meter (RE2-33005, Yamaden Co., Ltd., Tokyo, Japan). A cylindrical acrylic resin plunger (No. 56; 20 mm in diameter) was employed for compression. The instrumental settings were configured as follows: a load cell of 20 N, a compression speed of 10 mm/s, and a strain rate of 66.67%. Hardness stress (Pa), cohesiveness, and adhesiveness (J/m^3^) were calculated using the accompanying texture analysis software (Ver. 2.2, Yamaden Co., Ltd., Tokyo, Japan). Measurements were conducted in quintuplicate (*n* = 5) for each formulation, and the results are expressed as mean values.

#### 4.4.8. Sensory Evaluation

Sensory evaluation was conducted by 15 volunteers (4 males and 11 females) aged between 20 and 40 years, recruited from the university community. Prior to the evaluation, the panelists were briefed on the assessment attributes and instructed not to communicate with each other during the session to ensure independent scoring. The quality of the pudding gels was assessed using both analytical (discrimination) and preference (affective) tests on a 5-point bipolar scale (ranging from −2 to +2), following the protocol described in previous study [50,51].

For the analytical assessment, the intensity of specific attributes was scored from −2 to +2 as follows: color (light to dark), aroma (weak to strong), hardness (soft to firm), astringency (weak to strong), and smoothness (coarse to smooth). Regarding the affective test, preference for color, aroma, taste, texture, and overall liking was rated from −2 (unfavorable) to +2 (favorable), with a score of 0 representing “average”. To prevent flavor carryover, panelists were instructed to rinse their mouths with water between samples.

The study protocol was approved by the Ethical Review Committee for Research Involving Human Subjects at the Faculty of Human Sciences, Shimane University (Approval No. 231102). Prior to participation, the study’s objectives and procedures were explained to all subjects, and informed consent was obtained. Exclusion criteria included allergies to ingredients such as eggs or milk. Participants were assured of anonymity and the right to withdraw at any time. All samples were prepared under strict hygienic conditions.

#### 4.4.9. Determination of Soluble Tannin Content and Antioxidant Activity

Sample extraction was performed as follows: A 5 g portion of the pudding gel was weighed, mixed with 20 mL of 60% ethanol, and homogenized at 10,000 rpm for 2 min. The homogenate was then centrifuged at 1000 rpm for 3 min, and the supernatant was collected for analysis. For the control sample, as the supernatant appeared turbid, it was filtered through a 0.45 µm filter (Advantec Toyo Kaisha, Ltd., Tokyo, Japan) prior to use. The obtained supernatant was appropriately diluted with distilled water or buffer depending on the assay.

The STC of the pudding gels was determined using the Folin–Ciocalteu method as described in Section 4.3.2. The results were expressed as mg catechin equivalent per gram of fresh weight (FW) (mg CE/100 g FW).

The antioxidant activity was evaluated using the Hydrophilic Oxygen Radical Absorbance Capacity (H-ORAC) and 2,2-diphenyl-1-picrylhydrazyl (DPPH) radical scavenging assays. The H-ORAC assay was conducted following the method of Watanabe et al. [52] with minor modifications. The pudding extracts were diluted with 75 mM phosphate buffer (pH 7.4). The diluted sample, fluorescein, and 2,2′-azobis (2-amidinopropane) dihydrochloride (AAPH) were mixed in a 96-well plate. Fluorescence intensity (excitation: 485 nm, emission: 520 nm) was monitored every 2 min for 90 min at 37 °C using a microplate reader (SH-9000Lab, Corona Electric Co., Ltd., Ibaraki, Japan).

The DPPH radical scavenging activity was measured according to a previously reported method [53]. An aliquot of the diluted extract was mixed with a 2-(N-morpholino) ethanesulfonic acid (MES) buffer containing DPPH radicals. The mixture was incubated in the dark at room temperature for 20 min, after which the absorbance was measured at 540 nm. Both H-ORAC and DPPH values were expressed as µmol Trolox equivalent per gram of fresh weight (µmol TE/g FW).

### 4.5. Quantification of Allergenic Proteins (OVA, CAS, β-LG) in Pudding Gels

The concentrations of OVA from egg white, as well as CAS and *β*-LG from milk, were quantified using the FASPEK ELISA II^®^kit (Morinaga Institute of Biological Science, Inc., Yokohama, Japan). All assays were conducted in accordance with the manufacturer’s protocol.

Following the method described by Bugyi et al. [54], the FD pudding powder was utilized for the analysis. Protein extraction was performed by suspending the FD powder in the extraction buffer provided with the kit. Considering the high protein content of the samples, the standard extraction ratio was modified to mix 0.1 g of powder with 19.9 mL of extraction solution, followed by overnight shaking at 20 °C [4].

It was noted that the addition of CIS and YPF extracts increased the Brix of the pudding mixture, effectively diluting the protein content per unit weight of the FD powder. Consequently, analyzing a fixed mass (0.1 g) for all samples would lead to an underestimation of allergen concentrations in the treated groups. To correct for this dilution effect, the mass of FD powder weighed for extraction was adjusted based on the Brix values of the respective CIS and YPF extracts, ensuring that an equivalent amount of the pudding base was analyzed across all treatments.

### 4.6. Statistical Analysis

Data analysis was conducted using IBM SPSS Statistics for Windows, Version 28.0 (IBM Corp., Armonk, NY, USA). The STC values of the tannin extracts were compared using a *t*-test. For all other experimental data, multiple group comparisons were performed using a one-way analysis of variance (ANOVA), followed by Tukey’s honestly significant difference (HSD) post hoc test. Differences were considered statistically significant at *p* < 0.05.

## Figures and Tables

**Table 1:** Viscosity of pudding mixtures (mPa·s).

	Control	CIS		YPF	
		10%	50%	10%	50%
Viscosity (mPa·s)	6.93 ± 0.25 ^a^	25.89 ± 0.06 ^b^	94.87 ± 1.70 ^c^	11.90 ± 0.08 ^a^	117.87 ± 6.07 ^d^

Values are expressed as mean ± standard error (*n* = 3). Different letters in the table indicate statistically significant differences (*p* < 0.05). CIS, chestnut inner skin; YPF, young persimmon fruit. Control refers to pudding mixture without CIS or YPF. Samples labeled 10% and 50% indicate pudding mixtures where the respective percentage of distilled water (50 g) was replaced with CIS or YPF extract.

**Table 2:** Apparent density of the pudding mixture and pudding gels.

		Control	CIS		YPF	
			10%	50%	10%	50%
Apparent density (g/cm^3^)	Mixture	1.02 ± 0.01 ^c^	1.03 ± 0.03 ^c^	0.71 ± 0.03 ^a^	1.02 ± 0.07 ^c^	0.74 ± 0.08 ^b^
	Gel	1.09 ± 0.00 ^d^	0.99 ± 0.00 ^c^	0.84 ± 0.00 ^a^	1.12 ± 0.00 ^e^	0.88 ± 0.00 ^b^

Values are expressed as the mean ± standard error (*n* = 3). Different letters indicate statistically significant differences (*p* < 0.05). CIS, chestnut inner skin; YPF, young persimmon fruit. Control refers to samples without CIS or YPF. Samples labeled 10% and 50% indicate pudding mixtures or gels where the respective percentage of distilled water (50 g) was replaced with CIS or YPF extract.

## Data Availability

The data presented in this study are available in the article.
